# Mitigating Cardiotoxicity of Dendrimers: Angiotensin-(1-7) via Its Mas Receptor Ameliorates PAMAM-Induced Cardiac Dysfunction in the Isolated Mammalian Heart

**DOI:** 10.3390/pharmaceutics14122673

**Published:** 2022-12-01

**Authors:** Saghir Akhtar, Fawzi Babiker, Usman A. Akhtar, Ibrahim F. Benter

**Affiliations:** 1 College of Medicine, QU Health, Qatar University, Doha P.O. Box 2713, Qatar; 2 Departments of Physiology, Faculty of Medicine, Health Science Center, Kuwait University, Safat P.O. Box 24923, Kuwait; 3 Department of Mechanical and Chemical Engineering, College of Engineering, Qatar University, Doha P.O. Box 2713, Qatar; 4 Faculty of Medicine, Eastern Mediterranean University, Famagusta 99628, North Cyprus, Turkey

**Keywords:** PAMAM dendrimer, Ang-(1-7), cardiac ischemia/reperfusion, Mas receptor, surface chemistry, dendrimer generation, toxicity

## Abstract

Aim: The influence of the physiochemical properties of dendrimer nanoparticles on cardiac contractility and hemodynamics are not known. Herein, we investigated (a) the effect of polyamidoamine (PAMAM) dendrimer generation (G7, G6, G5, G4 and G3) and surface chemistry (-NH_2_, -COOH and -OH) on cardiac function in mammalian hearts following ischemia-reperfusion (I/R) injury, and (b) determined if any PAMAM-induced cardiotoxicity could be mitigated by Angiotensin-(1-7) (Ang-(1-7), a cardioprotective agent. Methods: Hearts isolated from male Wistar rats underwent regional I/R and/or treatment with different PAMAM dendrimers, Ang-(1-7) or its MAS receptors antagonists. Thirty minutes of regional ischemia through ligation of the left anterior descending coronary artery was followed by 30 min of reperfusion. All treatments were initiated 5 min prior to reperfusion and maintained during the first 10 min of reperfusion. Cardiac function parameters for left ventricular contractility, hemodynamics and vascular dynamics data were acquired digitally, whereas cardiac enzymes and infarct size were used as measures of cardiac injury. Results: Treatment of isolated hearts with increasing doses of G7 PAMAM dendrimer progressively exacerbated recovery of cardiac contractility and hemodynamic parameters post-I/R injury. Impairment of cardiac function was progressively less on decreasing dendrimer generation with G3 exhibiting little or no cardiotoxicity. Cationic PAMAMs (-NH_2_) were more toxic than anionic (-COOH), with neutral PAMAMs (-OH) exhibiting the least cardiotoxicity. Cationic G7 PAMAM-induced cardiac dysfunction was significantly reversed by Ang-(1-7) administration. These cardioprotective effects of Ang-(1-7) were significantly revoked by administration of the MAS receptor antagonists, A779 and D-Pro^7^-Ang-(1-7). Conclusions: PAMAM dendrimers can impair the recovery of hearts from I/R injury in a dose-, dendrimer-generation-(size) and surface-charge dependent manner. Importantly, PAMAM-induced cardiotoxicity could be mitigated by Ang-(1-7) acting through its MAS receptor. Thus, this study highlights the activation of Ang-(1-7)/Mas receptor axis as a novel strategy to overcome dendrimer-induced cardiotoxicity.

## 1. Introduction

Polyamidoamine (PAMAM) dendrimers, or “starburst dendrimers”, are nano-sized, spherical and highly-branched polymers that have important applications in nanomedicine, including as drug delivery carriers [1,2,3,4,5]. They can be synthesized by defined nanoparticle size, molecular architecture and surface charge or terminal functional group chemistry [2,3,5]. During PAMAM synthesis, sequential layers of radially repeating units are attached to a defined core (e.g., ethylenediamine), leading to progressive generations (G) of dendrimers with each consecutive generation having an increased molecular diameter and molecular weight due to the doubling of the number of surface functional groups compared to the previous generation [2,5]. Additionally, PAMAM dendrimers, can be produced with cationic amino-(-NH_2_), anionic carboxyl-(-COOH) or neutral hydroxyl-(-OH) terminal groups, all of which have been widely studied, including as potential drug delivery vectors [2,5,6]. Thus, PAMAMs with defined physicochemical properties, which are commercially available as a homologous series of low polydispersity polymers with increasing molecular weight and defined surface charge, readily lend themselves to structure activity relationship studies.

There is now growing evidence to suggest that beyond their ability to enhance drug delivery, PAMAM dendrimers have innate biological and toxicological actions that are highly dependent on their physicochemical properties (for recent review see [3]). Previously, we have shown that naked PAMAM dendrimers (without any drug cargo) can modulate key cell signaling networks, including those involving the epidermal growth factor receptor (EGFR), in a generation- (molecular weight) and surface charge- (functional group) dependent manner both in vitro and in vivo [7,8,9]. Due to passive accumulation of dendrimers within organs of the reticuloendothelial system, including the heart (for review see [3]), PAMAMs have been successfully used to deliver both small molecular weight drugs and gene-based therapies to cardiac tissue (e.g., [10,11,12,13]). However, little is known of their toxicological profile in the mammalian heart. 

We previously showed that systemically (intraperitoneally) administered PAMAM dendrimers could improve peripheral vascular function in vivo [8]. Thus, in a subsequent study, we hypothesized that PAMAMs might act similarly in the vasculature of the heart and thereby offer pharmacological benefit especially after cardiac ischemia-reperfusion injury (as would be required therapeutically after a “heart attack”). Contrary to our expectations, we recently reported that a G6 cationic PAMAM dendrimer actually impaired the ability of mammalian hearts to recover from ischemia-reperfusion injury ex vivo and in vivo [14]. Both systemic administration in vivo (daily i.p injections for 4 weeks) as well as acute, ex vivo administration directly to the isolated heart during reperfusion led to qualitatively similar effects on the heart with a cationic G6 PAMAM dendrimer [14]. However, the impact of other PAMAM dendrimer generations and surface chemical groups on cardiac function have not been studied. Thus, in the present study, we utilized the less time consuming and less costly approach of ex vivo (rather than in vivo) administration of dendrimers to isolated hearts during reperfusion to study the influence of PAMAMs of different generations and surface chemistries on cardiac recovery following ischemic injury, in the hope that lower generations or non-cationic PAMAMs might show some pharmacological benefit in acute cardiac ischemic injury. Another advantage of using the isolated perfused heart as a model is that perfusion with aqueous buffers largely avoids any potential blood complications, such as hemolysis or coagulation that might otherwise occur following direct, rapid intravenous administration of at least the cationic PAMAMs in vivo (for recent review see [3]).

Ischemic heart disease is one of the major health concerns globally [15,16,17]. Restriction of coronary blood supply (e.g., from atherosclerotic plaques) eventually leads to myocardial infarction (MI) and potentially death [18,19]. The extent of infarction and disease outcome can be mitigated to some extent by restoration of blood flow following ischemia [18,19]. However, reperfusion itself contributes to unavoidable damage in the myocardium, referred to as reperfusion or ischemia/reperfusion (I/R) injury [18,19]. Several pharmacological and mechanical cardioprotective procedures, including balloon-angioplasty, have been used to limit the devastating effects of I/R injury in the myocardium [20,21,22,23,24]. Pharmacologically, the use of thrombolytics such as streptokinase to degrade fibrin clots, or anticoagulation therapy with clopidogrel or aspirin, can also reduce thrombus size and restore coronary blood flow [25]. Several updated clinical and pre-clinical strategies for the pharmacological targeting of the underlying signaling pathways of MI have been reviewed recently [17]. Pharmacological conditioning of the heart (induced with drug(s)) can also provide protection akin to that of classical postconditioning [26,27,28]. However, we have been studying the cardioprotective effects of Angiotensin-(1-7), a member of the renin-angiotensin-aldosterone system (RAAS) that is crucial to the homeostasis of the cardiovascular system (for review see [29,30,31]).

Ang-(1-7) is a heptapeptide that generally acts via its Mas receptor to counter-regulate the functions of Angiotensin II (Ang II), the main peptide component of the RAAS. It can be synthesized by enzymatic cleavage of Ang II by Angiotensin converting enzyme 2 (ACE2)—which also doubles as the receptor for SARS-CoV2 in human cells [29,32]. The ACE2-Ang-(1-7)-Mas receptor axis of the RAAS promotes anti-oxidative stress, anti-inflammatory, anti-fibrotic and pro-vasodilatory effects that typically protect cardiovascular organs against various pathological injuries [29]. We have shown that Ang-(1-7)-mediated cardio-protection in animal models of diabetes and/or hypertension can occur via multiple mechanisms [33,34,35,36,37]. Thus, in this study, in addition to studying the impact of PAMAM dendrimer generation (G7, G6, G5, G4 and G3) and surface chemistry (-NH_2_, -COOH and -OH) on cardiac function, we sought to investigate whether Ang-(1-7) could mitigate the cardiotoxicity of high-generation cationic PAMAM dendrimers in an isolated, perfused rat heart model of I/R injury.

## 2. Materials and Methods

### 2.1. Materials

All materials and chemicals were purchased from Sigma Aldrich (St. Louis, Missouri, USA) unless stated otherwise. PAMAM dendrimers with an ethylenediamine core were produced by Dendritech (USA) and purchased from the Sigma Chemical Company (St. Louis, MO, USA). The properties of PAMAMs were characterized previously and we showed them to be mono-disperse structures [8]. The nominal physicochemical properties of PAMAM dendrimers and the doses used in this study are summarized in Table 1.

### 2.2. Animals and Procedures

**Figure 1:**
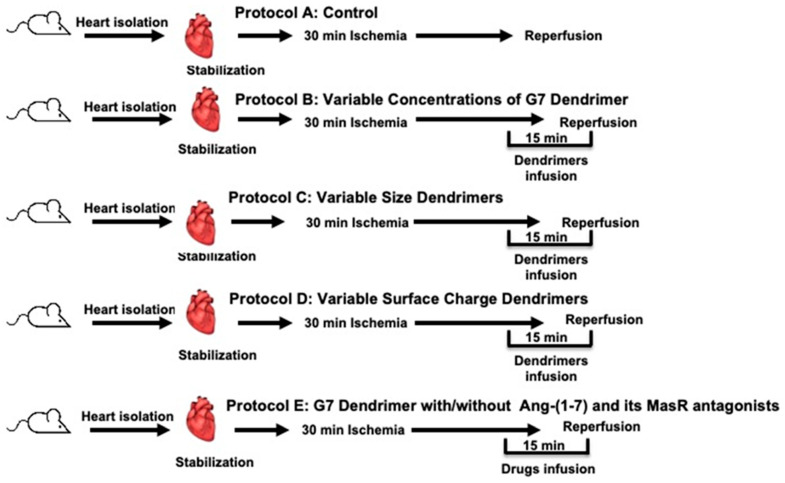
Schematic representation showing the experimental protocols used in the study (n = 8). A: Untreated ischemia-reperfusion control (C). B: Dose response relationship for the G7 PAMAM dendrimer. C: Effect of the dendrimer size (generation) on heart subjected to ischemia and reperfusion. D: Effect of the surface charge/chemistry of dendrimers on hearts subjected to ischemia and reperfusion. E: Effect of the G7 PAMAM dendrimer in the presence or absence of Ang-(1-7) and its Mas receptors antagonists on the effects of ischemia and reperfusion.

Male Wistar rats with body weights in the range of 250–350 g were obtained from the Kuwait University Animal Resources Centre. The study was approved by the Health Science Center, Kuwait University Animal Ethics Committee. The study was conducted as per the EU Directive 2010/63/EU for experiments in animals. All rats were kept under controlled conditions within a temperature range of 21–24 °C, a 12 h light/dark cycle (7 a.m.–7 p.m.) and a humidity of 50%. The rats were kept in plastic cages (2 rats/cage), with *ad libitum* access to food and water. The rats received anesthesia via an intraperitoneal (i.p) injection of a 60 mg/kg dose of sodium pentobarbital as well as an injection of the anti-coagulant, heparin (1000 U/kg body weight). Animal sacrifice was performed via cervical dislocation under general anesthesia. Surgery to isolate hearts [38] as well as cannulation and perfusion of the heart has been described by us previously [39]. Hearts underwent 30 min of regional ischemia through occlusion of the left anterior descending (LAD) branch of the coronary artery. We maintained a constant preload of 6 mmHg under basal controlled conditions and a constant perfusion pressure (PP) of 50 mmHg throughout the experimental procedures detailed in Figure 1. PP was measured using a Statham pressure transducer (P23 Db) and regulated electronically in the perfusion assembly (Module PPCM type 671 (Hugo Sachs Elektronik-Harvard Apparatus GmbH, Germany)) similar to that described previously [39].

### 2.3. Experimental Study Protocols

Rats were randomly assigned to 5 groups addressing 5 experimental protocols labelled as A-E (see Figure 1). Isolated hearts from rats (n = 8) in the first group (Protocol A) underwent 30 min ischemia and 30 min of reperfusion with no other treatment and served as controls (Figure 1). In protocol B, rat hearts (n = 8 for each dose) were subjected to 5 different concentrations of cationic G7 PAMAM dendrimer (1 µg, 5 µg, 7.5 µg, 10 µg or 20 µg/mL) to evaluate the dose-dependent effect of this dendrimer (Figure 1). In protocol C, we studied the cardiac effects of different dendrimer generations (molecular size) ranging from the smallest, G3 to G4, G5, G6, to the largest, G7, PAMAM dendrimers at a fixed concentration of 100 nM in isolated rat hearts (n = 8 for each dendrimer generation) (Figure 1, Protocol C). Protocol D was used to investigate the cardiac effects of the different surface chemistries (or charge) of the dendrimers whereby isolated hearts (n = 8 for each surface chemistry) were treated with either cationic G6, anionic G5.5 or neutral G6 PAMAM dendrimers at a fixed concentration of 100 nM (Figure 1, Protocol D). In protocol E, we investigated the effects of Ang-(1-7) and its Mas receptor antagonists on reversing G7 dendrimer cardiotoxicity. In addition to having control hearts subjected to I/R with no other treatment, other isolated rat hearts (N = 8 for each subgroup) were treated with either Ang-(1-7), cationic G7 PAMAM dendrimer; (G7) G7+(Ang-(1-7), G7+Ang-(1-7)+A779 (i.e., D-Ala^7^-Ang-(1-7) or G7+Ang-(1-7)+D-Pro (i.e., D-Pro^7^-Ang-(1-7)) (Figure 1, protocol E). All treatments were administered to isolated hearts 5 min before reperfusion and were continued during the first 10 min of reperfusion post I/R. Hearts undergoing I/R injury alone (without any other treatment) served as controls.

### 2.4. Assessment of Heart Function

The various cardiac function parameters relating to hemodynamics and contractility were determined during the period of stabilization (baseline) and after I/R injury as previously described [38,39,40]. Left ventricular (LV) dynamics were assessed through measuring the left ventricular (LV) end-diastolic pressure (LVEDP)- a measure of ventricular compliance that is typically elevated following acute myocardial infarction; and also the maximum developed pressure (DPmax) and LV contractility (+dP/dt or -dP/dt) parameters. The coronary vascular dynamics were determined through measuring coronary vascular resistance (CVR) and coronary flow (CF) as previously described [38,39,40].

### 2.5. Assessment of Cardiac Damage through Infarct Size Measurement and Determination of Cardiac Enzyme Levels

The size of LV infarcts was determined after staining with triphenyltetrazolium chloride (TTC) as described previously [41]. Images of infarcts from a given tissue slice were obtained using a Nikon camera and subsequently analyzed using Leica ImageJ (Wayne Rasb and National Institute of Health, USA), manually indicated on the image for each slice. The infarcted area (expressed as a percentage) on the image was calculated relative to total LV area. The cardiac enzymes, creatine kinase (CK) and lactate dehydrogenase (LDH) that were released in the coronary effluent during reperfusion were measured as described by us previously [42] as markers for cardiomyocyte injury.

### 2.6. Data Analysis

A two-way analysis of variance (ANOVA) followed by the least significant difference (LSD) post-hoc analysis of the data was performed using SPSS software. Comparisons between the data means of the different experimental groups and the mean for their respective controls was undertaken. All experimental data were presented as the mean ± standard error of the mean and statistically significance ascertained when values for *p* < 0.05.

## 3. Results

### 3.1. The Effects of Increasing Doses of Cationic G7 PAMAM Dendrimers on Cardiac Function Recovery following I/R Injury in Isolated Rat Hearts

**Figure 2:**
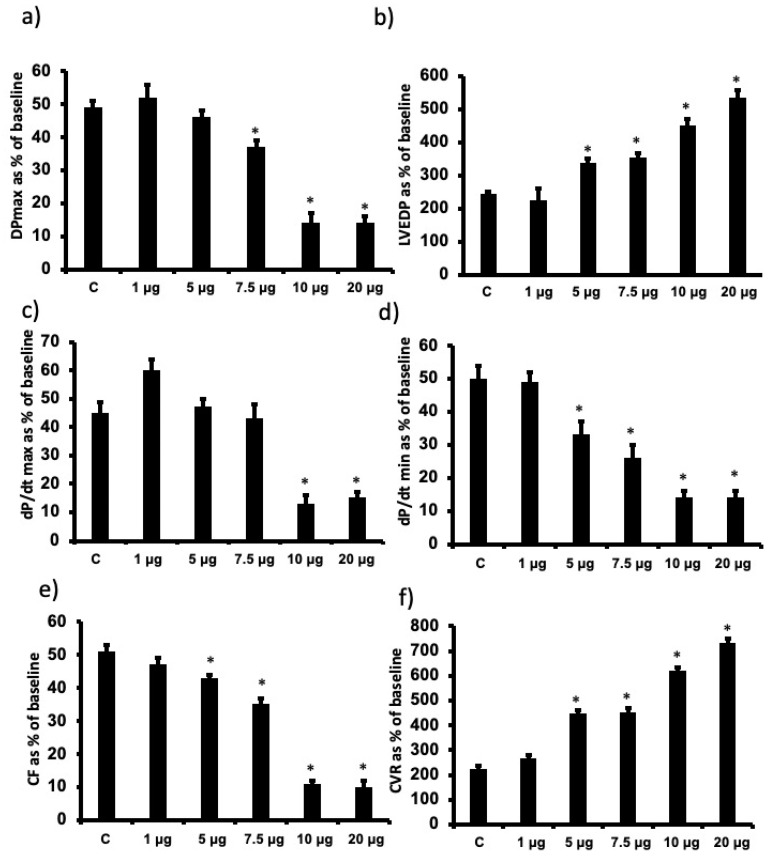
Dose-dependent recovery of cardiac function parameters following I/R upon acute administration of different doses of G7 cationic PAMAM (1.0 through 20 µg/mL). Percent recovery of cardiac function data (**a**–**f**) following I/R for left ventricle function (DPmax (**a**) and LVEDP (**b**)), contractility indices (+dP/dt (**c**) and −dP/dt (**d**) and coronary vascular dynamics (CF (**e**) and CVR (**f**)) are shown. The data were computed after 30 min reperfusion and expressed as the mean ± SEM. DPmax: maximum developed pressure; LVEDP: left ventricular end-diastolic pressure; CF: coronary flow; CVR: coronary vascular resistance. Control hearts, C = I/R alone. N = 8. Mean ± SEM. Asterix (*) indicates significant difference (*p* < 0.05) from controls.

In this study, the animal body weights (mean 300 ± 50 g) and heart size (1.5 ± 0.3 g) were not significantly different among the experimental groups studied. Regional ischemia followed by reperfusion caused a significant deterioration in the LV hemodynamics, contractility and coronary vascular dynamics compared to baseline data (*p* < 0.05). For example, % recovery in Pmax was only around 50% (Figure 2). Infusion of increasing doses of cationic G7 PAMAM dendrimer (1 µg, 5 µg, 7.5 µg, 10 µg or 20 µg/mL) at reperfusion resulted in a gradual increase in the deterioration of the LV hemodynamic, contractility and coronary vascular dynamics. For example, % recovery of Pmax gradually decreased with a statistically significant decline noted at doses of 7.5 µg/mL and above (*p* < 0.05) (Figure 2a). A similar deterioration was observed in LVEDP (*p* < 0.01), a measure of ventricular compliance (Figure 2b) as well as the other LV contractility parameters (+dp/dt; dp/dt) (Figure 2c,d). Hemodynamic parameters of CF and CVR also deteriorated with a significant decrease being observed at the higher doses (*p* < 0.05) (Figure 2e,f). For almost all cardiac function parameters, G7 PAMAM cardiotoxicity appeared to plateau at doses of 10 µg/mL and above (see Figure 2). For example, %R values for DPmax, +dp/dt; dp/dt plateaued at around 14%, whereas for CF, this value was around 10%. Similarly, a plateau was also observed for G7 PAMAM at doses of 10 µg/mL and above with LVEDP and CVR. 

### 3.2. The Influence of Cationic Dendrimer Generation on Cardiac Function Recovery following I/R Injury in Isolated Rat Hearts

**Figure 3:**
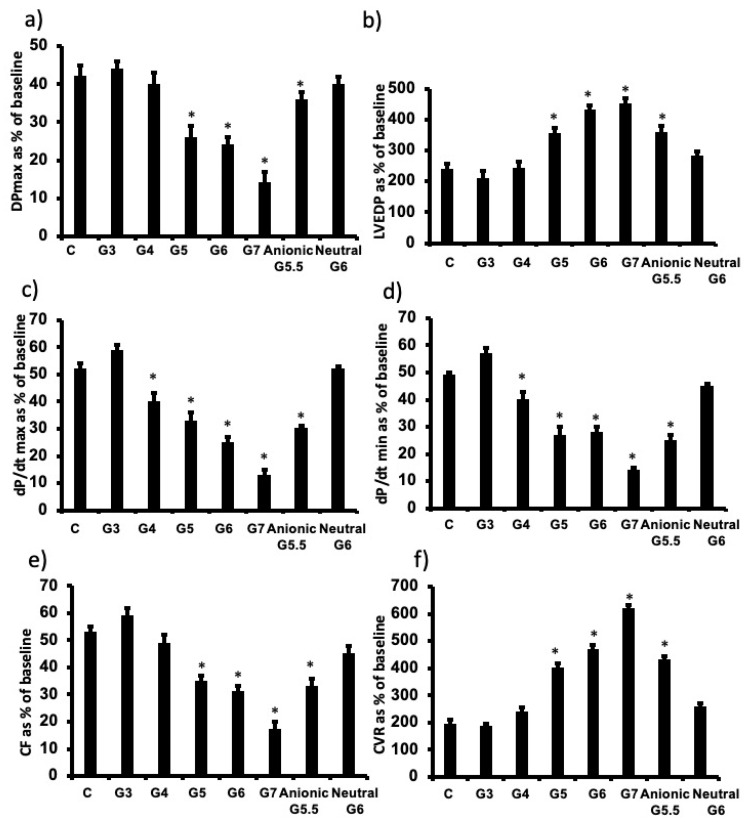
PAMAM-induced impairment in cardiac function is dependent on physicochemical properties of molecular size (generation) and surface charge of PAMAM dendrimers. Post I/R recovery in the left ventricle function (DPmax (**a**) and LVEDP (**b**)), contractility indices (+dP/dt (**c**) and –dP/dt (**d**)) and coronary vascular dynamics (CF (**e**) and CVR (**f**)) after treatment with various PAMAM dendrimer generations with variable molecular sizes (G3, G4, G5, G6, G7) or variable surface charge (cationic G6, anionic G5.5 or neutral G6). The data were computed after 30 min reperfusion and expressed as the mean ± SEM. DPmax: maximum developed pressure; LVEDP: left ventricular end-diastolic pressure; CF: coronary flow; CVR: coronary vascular resistance; G3: third generation PAMAM dendrimer; G4: fourth generation PAMAM; G5: fifth generation PAMAM; G6: sixth generation PAMAM; G7: seventh generation PAMAM G4. Control hearts, C = I/R alone. N = 8. Asterix (*) indicates significant difference (*p* < 0.05) from controls.

To determine the influence of PAMAM dendrimer generation (i.e., molecular size/weight) on cardiac function recovery post-ischemic injury, we infused a fixed dose (100 nM) of each of G7, G6, G5, G4 and G3 cationic (-NH_2_) PAMAM dendrimers. G3 generally exhibited little or no cardiac toxicity, whereas there was a gradual and significant decline in cardiac function recovery with progressively increasing dendrimer generation in terms of LV hemodynamics, contractility and coronary vascular dynamics (Figure 3). There was a 2-fold or greater decline in cardiac function from G4 to G7 cationic dendrimers as evidenced by the relative changes in %R values for Pmax, LVEDP, +dp/dt, dp/dt, CF and CVR parameters (see Figure 3). 

### 3.3. The influence of PAMAM Dendrimer Surface Chemistry on Cardiac Function Recovery after I/R Injury in Isolated Rat Hearts

To investigate the influence of dendrimer surface chemistry, we compared the cardiac effects of G6 neutral, G6 cationic and G5.5 anionic PAMAM dendrimers housing the following terminal chemical groups, respectively: hydroxyl- (-OH), amino- (–NH_2_) and carboxyl- (-COOH). Note that anionic PAMAM are only produced in half generations, hence the use of G5.5 -COOH (anionic) PAMAM for comparative purposes as the nearest molecular size to G6 that was used for cationic and neutral PAMAMs. Neutral G6 PAMAM exhibited little or no cardiotoxicity compared to charged PAMAMs, whereby cationic G6 PAMAM was more cardiotoxic than anionic G5.5 PAMAM (Figure 3). For example, following cardiac I/R injury, G6 cationic PAMAM reduced % recovery in Pmax by over 50%, whereas anionic reduced the same parameter by only around 25% and neutral surface chemistry had no effect (*p* < 0.05). Similar trends were observed for all other cardiac function parameters measured (LVEDP, +dp/dt, CF and CVR) except that for the dp/dt (max and min) function where both cationic and anionic PAMAMs compromised recovery to a similar degree (approximately 40–50%) (Figure 3).

### 3.4. PAMAM-Induced Cardiac Dysfunction Can Be Rescued by Ang-(1-7) in a Mas Receptor-Dependent Mechanism of Action

**Figure 4:**
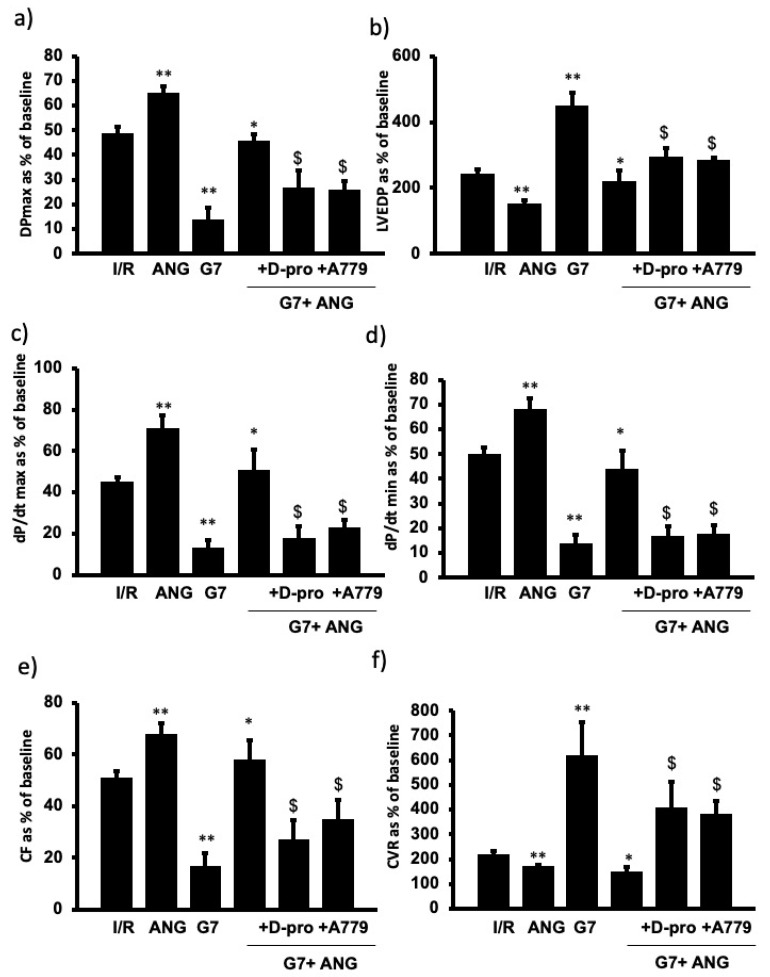
Ang-(1-7) via its Mas Receptor rescues cationic G7 PAMAM-induced impairment of cardiac function (**a**–**f**). Post I/R recovery in the left ventricle function (DPmax (**a**) and LVEDP (**b**)), contractility indices (+dP/dt (**c**) and −dP/dt (**d**)) and coronary vascular dynamics (CF (**e**) and CVR (**f**)) after treatment with G7 PAMAM in presence or absence of Ang-(1-7) and its Mas receptor blockers D-Pro and A779. The data were computed after 30 min reperfusion and expressed as the mean ± SEM. DPmax: maximum developed pressure; LVEDP: left ventricular end-diastolic pressure; CF: coronary flow; CVR: coronary vascular resistance; Ang-(1-7): angiotensin-91-7); D-Pro: Ang-(1-7) selective antagonist; A779: Ang-(1-7) selective antagonist (see Section 2). Double Asterix ** refers to significant difference (*p* < 0.05) compared to control I/R alone and single Asterix * refers to significant difference (*p* < 0.05) compared G7 values. Dollar sign ($) indicates significant difference (*p* < 0.05) compared to G7 + Ang-(1-7).

**Figure 5:**
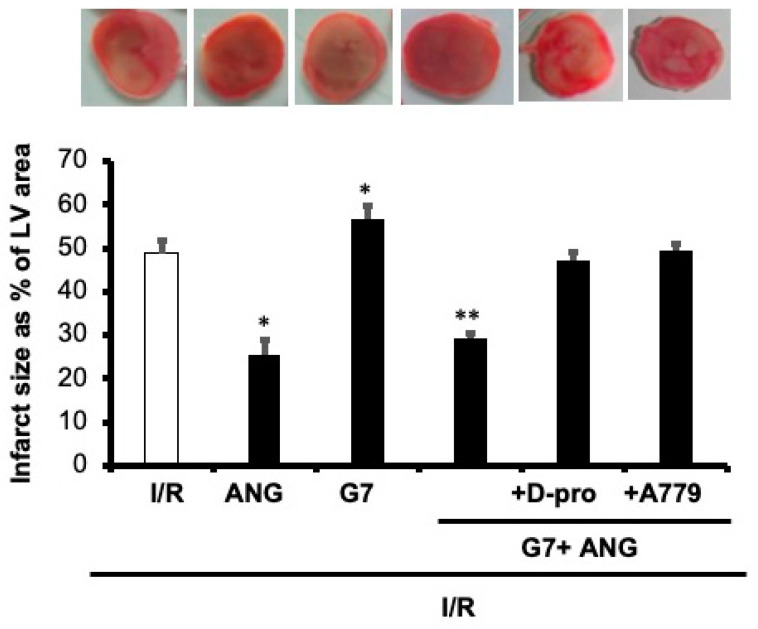
Ang-(1-7) via its Mas Receptor rescues cationic G7 PAMAM-induced myocardial infarction. Infarct size post-I/R injury was determined after treatment with G7 PAMAM in the presence or absence of Ang-(1-7) and its Mas receptor blockers D-Pro and A779 (n = 4). Top panel: representative 2,3,5-triphenyl-2H-tetrazolium chloride-stained heart slices for each treatment condition. Bottom Panel: measured infarct size, normalized to the LV area, in isolated rat hearts at the end of reperfusion. C: control; G7: seventh generation cationic PAMAM dendrimer; Ang-(1-7): angiotensin-1-7; D-Pro: Ang-(1-7) selective antagonist; A779: Ang-(1-7) selective antagonist. Single Asterix * refers to significant difference (*p* < 0.05) compared to control I/R alone and double Asterix ** to significant difference (*p* < 0.05) compared to G7 values.

To examine the potential beneficial effect of Ang-(1-7) in protecting the rat heart against the cardiotoxic effects of G7 PAMAM dendrimer, we administered this drug during reperfusion in the absence or presence of the G7 PAMAM dendrimer and/or its Mas receptor blockers, A-779 or D-Pro (see Section 2. The infusion of G7 PAMAM dendrimer (10 μg/mL) induced a significant deterioration in almost all cardiac function parameters compared to control (Figure 4). Ang-(1-7) treatment significantly improved (*p* < 0.001) recovery of DPmax, LVDP and LV contractility from I/R injury alone or upon treatment with the cationic G7 PAMAM dendrimer (Figure 4a–d). A similar cardioprotective effect was noticed on coronary vascular dynamics as the deterioration caused by I/R alone or following G7 PAMAM dendrimer treatment was rescued by adjunct administration of Ang-(1-7) (*p* < 0.001) (Figure 4e,f). The cardioprotection afforded by Ang-(1-7) was largely revoked in the presence of its Mas receptor antagonist, A-779 or D-Pro (Figure 4), implying that the beneficial effects of Ang-(1-7) in mitigating dendrimer-induced cardiotoxicity were mediated, at least in part, via its Mas receptor. These results from cardiac function recovery studies were confirmed by evaluation of the cardiac enzyme levels and the infarct size (see Table 2). Ang-(1-7) treatment largely neutralized both the I/R injury and cationic G7 PAMAM-induced cardiac damage as evidenced by decreased CK and LDH enzyme levels and myocardial infarct size (*p* < 0.05 and *p* < 0.01 respectively) (see Table 2 and Figure 5).

## 4. Discussion

**Figure 6:**
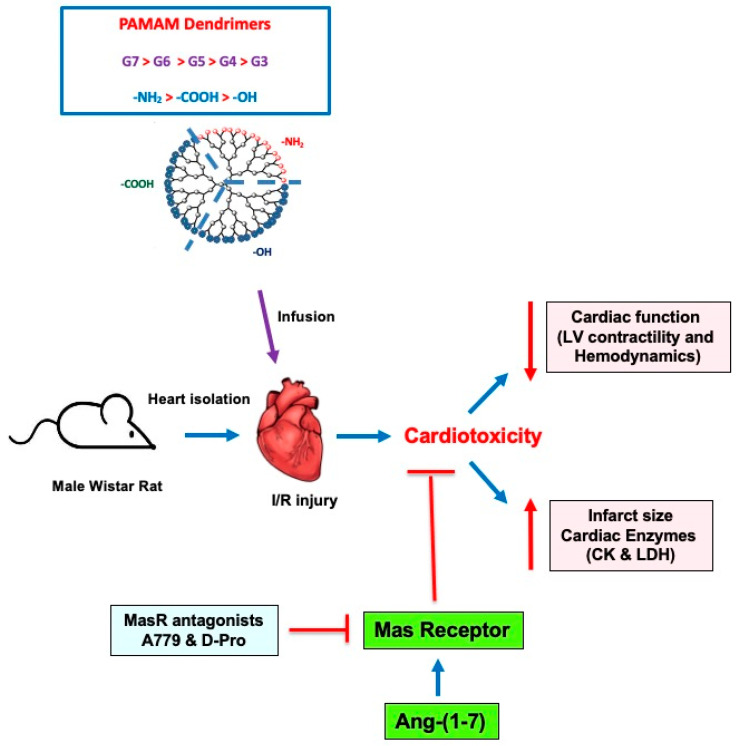
A schematic summary of the impact of different PAMAMs in the mammalian heart and the ability of Ang-(1-7) to mitigate their cardiotoxicity. In isolated rat hearts subjected to I/R injury, administration of PAMAM dendrimers exacerbated recovery of cardiac function in terms of LV contractility and hemodynamics parameters as well by increasing infarct size and cardiac enzyme levels (LDH and CK)—hallmarks of cardiac damage and toxicity. These effects of PAMAMs were dependent on dendrimer generation (G7 > G6 > G5 > G4 > G3) and surface charge ((-NH_2_ (cationic) > -COOH (anionic) > -OH (neutral)). The adjunct administration of Ang-(1-7) rescued the cardiotoxicity caused by cationic PAMAM dendrimers. The beneficial effects of Ang-(1-7) were revoked by two Mas receptor (MasR) antagonists (A779 and D-Pro), confirming that Ang-(1-7) actions were, at least in part, mediated through MasR. Thus, Ang-(1-7) may represent a viable strategy to mitigate the cardiotoxicity of PAMAM dendrimers.

PAMAM dendrimers have been proposed to have multiple roles in clinical nanomedicine including as drug delivery vectors [3]. However, the toxicological profiles of these dendrimers in specific organs and tissues are not fully elucidated. Indeed, naked dendrimer nanoparticles (without any drug cargo) are known to exert biological and toxicological actions of their own in several biological systems [3] but their direct biological/toxicological impact on the mammalian heart is understudied. We previously showed that a cationic G6 dendrimer administered chronically over four weeks to healthy and diabetic rats could partially impair recovery of heart function following I/R injury [14]. However, the influence of the different PAMAM dendrimer physiochemical properties, such as generation (molecular size/number of surface groups) and surface charge, on cardiac contractility and hemodynamics functions are not known. Thus, we examined the effect of cationic PAMAM dendrimer generation (G7, G6, G5, G4 and G3) and surface chemistry (-NH_2_ (cationic), -COOH (anionic) and -OH (neutral)) on recovery of cardiac function parameters in mammalian hearts after I/R injury. The key findings of this study are that cationic G7 PAMAM dendrimer dose-dependently impaired cardiac contractility and hemodynamics in the isolated, perfused rat heart and that impairment of cardiac function was generally dependent on the key physicochemical properties of dendrimer generation (G7 > G6 > G5 > G4 > G3 that had little or no effect) and surface chemistry (cationic > anionic > neutral that had little or no effect)). Importantly, we further showed that cardiotoxicity of cationic PAMAM dendrimer nanoparticles could be mitigated by co-administering the cardioprotective agent, Ang-(1-7). Mechanistically, the cardio-protection afforded by Ang-(1-7) in reversing PAMAM-induced cardiac injury occurred, at least in part, via its Mas receptor, as two different Mas receptor antagonists largely revoked the cardio-protection afforded by this heptapeptide (see also Figure 6). Thus, our studies show, for the first time, that the physiochemical properties of dendrimer generation (molecular size) and surface charge are important determinants of PAMAM cardiotoxicity, and critically, that administration of Ang-(1-7) may represent a novel strategy to mitigate cardiotoxicity of PAMAM dendrimers and possibly other nanoparticle drug delivery systems.

The fact that we found nanoparticle surface charge to be a key determinant of PAMAM-mediated cardiac dysfunction is consistent with our own studies on the biological and toxicological effects of PAMAMs in other systems [8,9]. Charged PAMAMs especially those bearing cationic surface chemistry generally exhibit a greater cellular toxicity than neutral PAMAMs [6]. The greater cardiotoxicity observed with cationic PAMAMs in this study is further supported by the discovery that cationic PAMAMs, compared to their neutral counterparts, reportedly exhibit greater biodistribution to the heart [43]. Concerning the heart, there is also evidence in the literature suggesting that PAMAMs preferentially accumulate in ischemic cardiac tissue following I/R injury compared to the normal non-diseased myocardium [44], implying that the ischemic heart may be more prone to the adverse toxicological effects of charged PAMAM dendrimers compared to healthy heart tissue, though this requires further study and validation experimentally. The impairment of cardiac function recovery was also dependent on dendrimer generation (see Figure 3). G7 dose-dependently compromised cardiac contractility and hemodynamics following I/R injury and the cardiotoxicity of cationic PAMAMs markedly decreased with progressively lower generations, with G3 having little or no effect on cardiac functional recovery post-I/R injury. These data implied that lower generation PAMAMs, even those bearing cationic surface charges, may be safer to use in vivo than the higher generation cationic PAMAMs that exhibited marked cardiotoxicity. The dependency of cardiac function on dendrimer generation may be suitably explained by the fact that higher generations have progressively greater positively charged surface groups that facilitate increased cellular accumulation and thus result in greater biological and toxicological actions (for reviews see [3,45]). This is additional to the general phenomenon of nanoparticles administered systemically, passively bio-distributing to organs of the reticulo-endothelial system that include the heart [3,45].

The mechanism by which PAMAMs result in impairment of cardiac function recovery from I/R injury was not studied here and is a potential limitation of our study. However, the outcomes of several biological and toxicological studies from our group, as well as others, do offer some insights [7,8,9,46,47,48,49,50,51,52,53]. Collectively, these studies suggest that beyond their role as drug delivery agents, PAMAM dendrimers house the potential to modulate important cellular genes and protein signaling networks in vitro and in vivo, which can lead to increased oxidative stress-induced injury and apoptosis. PAMAMs can also interfere with key receptor signaling networks such as those involving the epidermal growth factor receptor (EGFR) family of receptor tyrosine kinases (RTKs) (for review see [3]). EGFR RTKs have immense importance in physiological functions such as cell proliferation, growth, differentiation, motility, migration and apoptosis [54,55]. Dysregulated EGFR signaling has long been established to be associated with cancer [54,56] but our previous studies have also highlighted its importance in cardiovascular pathology (for review see [30]). For example, we previously showed that EGFR signaling mediates cardiac preconditioning [57] and is also critical for recovery of hearts post-I/R injury [58,59], implying that EGFR likely represents an important component of the “salvage pathways” that are necessary for the heart to recover from I/R injury. We have also demonstrated that administration of PAMAM dendrimers can block EGFR signaling in vivo and in vitro [8,48]. Thus, it is tempting to speculate that impaired cardiac function resulting from PAMAM exposure, especially that induced by high generation cationic PAMAMs, is likely mediated through a blockade of EGFR signaling—a key salvage pathway known to be involved in recovery of hearts post-I/R injury. Alternatively, cardiac function impairment, especially in CF and CVR, might occur via dendrimer-induced clot formation and subsequent occlusion of the coronary vasculature, as it has been shown that rapid i.v. administration of cationic PAMAM nanoparticles can induce hemolysis and blood coagulation [60]. However, this phenomenon is thought to be less likely when there is a slower biodistribution of dendrimer into the blood such as following i.p. administration [3,8,61]. Given the fact from our previous studies that even i.p administration of a cationic G6 PAMAM, where blood coagulation should be minimal, impaired cardiac recovery from I/R injury [14] and PAMAMs actually had beneficial effects in blood vessels by preventing diabetes-induced vascular dysfunction and remodeling [8], implies that it is more likely that these dendrimers inhibit key cardiac survival or salvage signaling cascades. However, chronic i.p administration of a cationic PAMAM (G4) dendrimer was reported to attenuate cardiac mitochondrial function [62], implying that, mechanistically, these dendrimers may also impair heart function through a dysregulation of mitochondrial function. Although these hypotheses need further study, strategies that mitigate the toxicity of PAMAMs will be useful in the potential use of PAMAMs as drug delivery systems in cardiovascular medicine. 

Our current study suggests that the cardiotoxicity of high generation cationic PAMAMs can be circumvented by using lower generation cationic PAMAMs (e.g., G3) or PAMAMs with a neutral surface charge as these had the least effect on cardiac function. However, higher generation PAMAMs may be more desirable in some applications, e.g., for potential drug targeting to the ischemic heart to take advantage of their higher and selective accumulation in the heart (see discussion above). PEGylation and partial masking of charges are other possible approaches to off-set PAMAM toxicity (see [6] for recent review). However, in this study, we sought to determine if the adjunct delivery of a cardioprotective agent, Ang-(1-7) might mitigate cardiotoxicity of a high generation (G7) cationic dendrimer. We have previously shown that Ang-(1-7) protects hearts from cardiac ischemia injury [36,63] and mediates the beneficial effects of pacing post-conditioning [37], most likely through multiple mechanisms including anti-inflammatory, anti-oxidative stress and pro-vasodilatory actions (for reviews see [29,30,31]). In the present study, adjunct administration of Ang-(1-7) largely ameliorated the detrimental cardiac effects of G7 PAMAM dendrimer, an effect that was, at least partially, revoked by selective Mas receptor inhibitors, A-779 or D-Pro, confirming that Ang-(1-7)/Mas receptor axis was involved in mediating cardioprotection. As to the possible downstream effectors of Ang-(1-7)-mediated cardioprotection, likely candidates include the inhibition of the pro-inflammatory transcription factor, NF-kB [33,64], the oxidative stress-inducing NADPH-oxidases [36] and increased NO synthesis [37]. The latter would also facilitate coronary vessel vasodilation that might reduce or prevent any vessel occlusion that might be occurring as part of PAMAM-mediated cardiotoxicity. Indeed, a NO-releasing drug conjugated to a G4 PAMAM dendrimer improved cardiac function post I/R injury in an isolated, perfused rat heart [11]. Furthermore, since PAMAMs are known to induce oxidative stress, apoptosis and in some cases proinflammatory responses (for review see [6]), it is possible that Ang-(1-7) rescues PAMAM-induced cardiac dysfunction through a correction or counter-regulation of the pathways negatively affected by PAMAM dendrimers. Alternatively, the cardioprotective effects of Ang-(1-7) may occur via pathways independent of those adversely impacted by PAMAMs, and clearly both possibilities require further experimental study. The concept that Ang-(1-7) may serve as a novel therapeutic agent in mitigating cardiovascular toxicity of xenobiotics is further supported by the recent finding that Ang-(1-7) could reduce rat aortic arch dysfunction induced by the anti-cancer agent doxorubicin [65]. Additionally, Ang-(1-7) mitigated renal injury induced by gentamicin, an aminoglycoside antibiotic [66]. Thus, we propose that co-administration of Ang-(1-7) may represent a novel strategy to mitigate cardiotoxicity of nanoparticles in general as well as PAMAM dendrimers as described in our present study. As Ang-(1-7) is a peptide drug, to improve its biological stability and delivery in vivo, it can be formulated with cyclodextrins or even PAMAM delivery systems, which have been shown to be effective [66,67,68,69]. Furthermore, in clinical trials, Ang-(1-7) appears to be well tolerated and safe to use in humans [70,71].

Though not studied here, other cardioprotective drugs entrapped within or conjugated to the outer surface of PAMAM dendrimer nanoparticles might afford similar cardioprotection to Ang-(1-7). For example, an agonist of the A3 adenosine receptor, an important player in post-I/R cardiac recovery pathways, when conjugated to a G4 PAMAM dendrimer, led to cardioprotective effects in isolated hearts subjected to I/R injury [13]. Similarly, polymer nanoparticles laden with the cargo of antioxidants or anti-inflammatory agents, including curcumin or resveratrol, were also cardioprotective in animal models [72,73]. Therefore, by careful selection of dendrimer-drug combinations, or simple adjunct administration of effective cardioprotective agents such as Ang-(1-7), PAMAM dendrimers could conceivably be converted from potentially cardiotoxic to cardio-safe or even cardioprotective agents. Indeed, such approaches might be essential for mitigating dendrimer toxicity and for PAMAM-containing nanomedicines to meet the required safety profile for use in the clinic.

## 5. Conclusions

Administration of G7 PAMAM dendrimer dose-dependently attenuated cardiac contractility and coronary vascular dynamic functions following I/R injury. Impairment of cardiac function recovery correlated with the physicochemical properties of dendrimers with a strong influence of both surface charge and molecular size or generation. Neutral PAMAMs and low generation cationic PAMAMs (e.g. G3) appeared to have little or no effect on cardiac function and appeared safe for potential pre-clinical and clinical applications. Importantly, Ang-(1-7) mitigated cationic G7 PAMAM-induced cardiac dysfunction via a pathway involving its Mas receptor. We therefore propose that the adjunct use of Ang-(1-7) may represent a novel strategy to mitigate cardiotoxicity of cationic PAMAM nanoparticles. Our findings are therefore deemed highly important in further understanding the toxicology of dendrimers in the mammalian heart and, by identifying a novel strategy for mitigating their cardiotoxicity, may facilitate a broader and safer use of cationic PAMAMs in clinical nanomedicine.

## Figures and Tables

**Table 1:** List of PAMAM dendrimers, their nominal physicochemical properties and dosages used in this study.

Terminal Surface Chemistry ^$^	Surface Charge	Generation ^$^	Molecular Weight (Da) ^$^	Diameter (nm) ^$^	No. of Surface Groups ^$^	Dose (s) Administered in Isolated Rat Heart
-NH_2_	Cationic	3	6909	3.6	32	100 nM
-NH_2_	Cationic	4	14,215	4.5	64	100 nM
-NH_2_	Cationic	5	28,826	5.4	128	100 nM
-NH2	Cationic	6	58,048	6.7	256	100 nM
-NH_2_	Cationic	7	116,493	8.1	512	100 nM and for dose-dependent studies: 1 µg, 5 µg, 7.5 µg, 10 µg or 20 µg/mL
-OH	Neutral	6	58,304	NA	256	100 nM
-COOH	Anionic	5.5	52,913	NA	256	100 nM

^$^ Manufacturer provided information. NA, Information not made available. Anionic PAMAM dendrimers are produced as half-generations. All dendrimers had an ethylenediamine core structure.

**Table 2:** Effects of Ang-(1-7) and its Mas receptor antagonists, ischemia/reperfusion (I/R) and cationic G7 PAMAM dendrimer on cardiac enzymes levels. CK = Creatinine kinase; LDH = lactate dehydrogenase. G7: seventh generation cationic PAMAM dendrimer; Ang-(1-7): angiotensin-1-7; D-Pro and A779 are Ang-(1-7) selective antagonists. Asterix * refers to significant difference (*p* < 0.05) compared to control I/R alone.

Treatment	CK (IU/L)	*p* Value	LDH (IU/L)	*p* Value
I/R	35.77 ± 0.46	-	28.27 ± 1.12	-
Ang-(1-7)	26.97 ± 1.43 *	0.001	20.24 ± 0.39 *	0.001
G7	46.26 ± 1.39 *	0.022	40.41 ± 0.99 *	0.01
G7 + Ang-(1-7)	28.34 ± 0.86 *	0.002	21.73 ± 0.73 *	0.001
G7 + Ang-(1-7) + DPro	35.22 ± 1.21	0.709	29.40 ± 0.76	0.294
G7 + Ang-(1-7) + A779	35.32 ± 0.96	0.633	29.33 ± 0.39	0.383

## Data Availability

The data presented in this study are available on request from the corresponding author.
